# Caspase-2 mediates a *Brucella abortus* RB51-induced hybrid cell death having features of apoptosis and pyroptosis

**DOI:** 10.3389/fcimb.2013.00083

**Published:** 2013-11-27

**Authors:** Denise N. Bronner, Mary X. D. O'Riordan, Yongqun He

**Affiliations:** ^1^Department of Microbiology and Immunology, University of Michigan Medical SchoolAnn Arbor, MI, USA; ^2^Unit for Laboratory Animal Medicine, University of Michigan Medical SchoolAnn Arbor, MI, USA; ^3^Comprehensive Cancer Center, University of Michigan Medical SchoolAnn Arbor, MI, USA

**Keywords:** proinflammatory, caspase-2, programmed cell death, mitochondrial dysfunction, macrophage, Brucella

## Abstract

Programmed cell death (PCD) can play a crucial role in tuning the immune response to microbial infection. Although PCD can occur in different forms, all are mediated by a family of proteases called caspases. Caspase-2 is the most conserved caspase, however, its function in cell death is ill-defined. Previously we demonstrated that live attenuated cattle vaccine strain *Brucella abortus* RB51 induces caspase-2-mediated and caspase-1-independent PCD of infected macrophages. We also discovered that rough attenuated *B. suis* strain VTRS1 induces a caspase-2-mediated and caspase-1-independent proinflammatory cell death in infected macrophages, which was tentatively coined “caspase-2-mediated pyroptosis”. However, the mechanism of caspase-2-mediated cell death pathway remained unclear. In this study, we found that caspase-2 mediated proinflammatory cell death of RB51-infected macrophages and regulated many genes in different PCD pathways. We show that the activation of proapoptotic caspases-3 and -8 was dependent upon caspase-2. Caspase-2 regulated mitochondrial cytochrome *c* release and TNFα production, both of which are known to activate caspase-3 and caspase-8, respectively. In addition to TNFα, RB51-induced caspase-1 and IL-1β production was also driven by caspase-2-mediated mitochondrial dysfunction. Interestingly, pore formation, a phenomenon commonly associated with caspase-1-mediated pyroptosis, occurred; however, unlike its role in *S. typhimurium*-induced pyroptosis, pore formation did not contribute to RB51-induced proinflammatory cell death. Our data suggest that caspase-2 acts as an initiator caspase that mediates a novel RB51-induced hybrid cell death that simulates but differs from typical non-proinflammatory apoptosis and caspase-1-mediated proinflammatory pyroptosis. The initiator role of the caspase-2-mediated cell death was also conserved in cellular stress-induced cell death of macrophages treated with etoposide, naphthalene, or anti-Fas. Caspase-2 also regulated caspase-3 and -8 activation, as well as cell death in macrophages treated with each of the three reagents. Taken together, our data has demonstrated that caspase-2 can play an important role in mediating a proinflammatory response and a hybrid cell death that demonstrates features of both apoptosis and pyroptosis.

## Introduction

Programmed cell death (PCD) is a crucial process initiated by the host in response to cellular stress and microbial infections. PCD can occur in a variety of ways (Galluzzi et al., 2012). Apoptosis, pyroptosis, and necroptosis are three pathways of PCD that can occur during microbial infections with substantially different outcomes. Apoptosis is a silent PCD due to a lack of cytokine secretion (Elmore, 2007). Although the plasma membrane remains intact, apoptotic bodies can bleb off and be phagocytosed by other phagocytic cells in the surrounding area. Pyroptosis or “*death by fire*,” is inflammatory PCD typically mediated by caspase-1 (Bergsbaken et al., 2009). Caspase-1 processes proinflammatory cytokines (IL-1β and IL-18), and secretion of these cytokines requires pore formation in the plasma membrane, which leads to cell swelling and eventually lysis. Necroptosis is a newly identified type of PCD that includes a proinflammatory response as well as loss of plasma membrane integrity (Vandenabeele et al., 2010). In contrast to apoptosis and pyroptosis, serine/threonine kinases, RIP1 and RIP3, mediate necroptosis. In addition, necroptosis leads to the release of intracellular contents; mostly damage associated molecular patterns (DAMPs).

Many microbes can induce cell death during infection and dissemination (Gao and Abu Kwaik, 2000). Avirulent *Mycobacterium* induces apoptosis in macrophages (Fratazzi et al., 1999). Neighboring uninfected macrophages, upon phagocytosis, killed *Mycobacterium* in apoptotic bodies released by *Mycobacterium*-infected macrophages. In addition, apoptotic blebs from bacterially infected cells induce a T_H_17 response. In contrast, *Shigella* and *Salmonella*-induced pyroptosis leads to the release and exposure of bacteria to reactive oxygen species (ROS) and neutrophils (Hilbi et al., 1998; Brennan and Cookson, 2000; Miao et al., 2010). In addition to bacterial pathogens, parasitic and viral pathogens such as *Trypanosoma cruzi* and Vaccinia virus also have the ability to induce apoptosis and necroptosis, respectively (Cho et al., 2009; Duaso et al., 2011). The outcomes of necroptosis are an increase in cytokine secretion and leukocyte infiltration as well as ROS production. As illustrated from previous studies, PCD can play an important role in controlling microbial infections. Meanwhile, many pathogens can inhibit these PCD pathways in various approaches. For example, virulent wild type (WT) *Brucella* strains typically inhibit PCD of infected macrophages (Chen and He, 2009; Chen et al., 2011; Li and He, 2012). Elucidating the PCD mechanism induced or inhibited by such pathogens is critical to uncovering mechanisms of pathogenesis, as well as protective immunity.

The main executors of the PCD process are caspases, which are divided into two groups: initiators and effectors. Initiator caspases activate effector caspases via cleavage whereas effector caspases initiate cell death by cleaving various downstream apoptotic proteins. *C. elegans* has a single caspase, Ced-3, that mediates all cell death. Of 13 caspases existing in mammalian systems, caspase-2 has the highest sequence homology with Ced-3 (Hengartner, 1997; Geng et al., 2009). Caspase-2 plays important biological roles from oocyte development to aging control, and in intermediary development stages including DNA damage repair, tumor prevention, and infection control (Guo et al., 2002; Ho et al., 2009; Shi et al., 2009; Bouchier-Hayes and Green, 2012). Caspase-2 can play different roles due to its unique domain structure, which resembles an initiator and effector caspase. It contains a caspase activation and recruitment domain (CARD) which is required for auto-activation and binding to other molecules. Caspase-2 also contains a cleavage site (Hofmann et al., 1997) which resembles that of the effector caspase-3 (Talanian et al., 1997). These factors make the classification of caspase-2 difficult. Caspase-2-deficient mice develop without an overt phenotype although only mild apoptotic defects in oocyte and neuron developments were exhibited, suggesting that the function of caspase-2 is largely redundant for cellular homeostasis during development (Bergeron et al., 1998). Caspase-2 has been shown to be instrumental in bacterial infections. Caspase-2 played a role in both caspase-1-dependent and -independent apoptosis of macrophages infected with *Salmonella* (Jesenberger et al., 2000). The various and often controversial roles of caspase-2 in different organisms and experimental conditions have been documented and discussed (Troy and Ribe, 2008; Kitevska et al., 2009). The role of caspase-2 in regulating cell death and the exact mechanism remain unclear.

We previously demonstrated that rough attenuated *Brucella abortus* strain RB51 induces caspase-2-mediated, caspase-1-independent apoptotic and necrotic cell death (Chen and He, 2009). As a licensed cattle vaccine strain, RB51 is able to induce IFNγ and CD8^+^ T cell mediated cytotoxicity in mice (He et al., 2001). Unlike its virulent counterparts, RB51 does not replicate in macrophages but it induces robust caspase-2-mediated apoptotic and necrotic cell death (Chen and He, 2009). In addition, RB51 induces cell death in dendritic cells (Li and He, 2012). However, the caspase-2-mediated RB51-induced cell death pathway is largely unknown. Previously, we showed that caspase-2 activation as well as decrease of the mitochondrial membrane potential occurred in dying macrophages infected with RB51 (Chen and He, 2009). These characteristics would suggest that apoptosis via the mitochondria-driven intrinsic pathway was the cell death mechanism. We also showed that rough attenuated *B. suis* strain VTRS1 induces caspase-2-mediated proinflammatory cell death, which we tentatively named “caspase-2-mediated pyroptosis” (Chen et al., 2011). It is likely that RB51 also induces proinflammatory response that differs inherently from non-proinflammatory apoptosis. How RB51 induces cell death remains unclear.

Here we investigated which PCD mechanism was responsible for RB51-induced cell death in macrophages. We found that RB51-infected macrophages exhibited mitochondrial dysfunction, activation of the caspase cascade (caspase-3 and caspase-8), IL-1β and TNFα secretion, and pore formation in the plasma membrane—all of which were dependent upon caspase-2. In addition to infection, we found that caspase-2 also mediated cell death as well as caspase-3 and -8 activation in macrophages treated with etoposide, naphthalene, and anti-Fas. These results illustrate that RB51-induced caspase-2-mediated macrophage cell death is unique in that it exhibits characteristics of both non-proinflammatory apoptosis and caspase-1-mediated proinflammatory pyroptosis.

## Results

### Caspase-2 mediates RB51-induced cell death via intrinsic and extrinsic apoptosis pathways

Our previous experiments using a chemical inhibitor and siRNA demonstrated that caspase-2 mediates RB51-induced macrophage cell death (Chen and He, 2009). To confirm the caspase-2 mediated cell death, WT and caspase-2 deficient (*casp*2^−/−^) bone-marrow derived macrophages (BMDMs) were infected with RB51, and cell death was assessed by Annexin V/propidium iodide (PI) staining and lactate dehydrogenase (LDH) release. The RB51 infection was confirmed to induce strong macrophage cell death as shown at 24 h post-infection (h.p.i., Figures S1A,B). In contrast, *casp2*^−/−^ BMDMs exhibited little or no cell death throughout infection and only reached 8.5 ± 4.7% and 8.9 ± 5.1% at 24 h.p.i. in Annexin V/PI and LDH release assays, respectively. These results confirmed that RB51-induced cell death is mediated by caspase-2. We also assessed if there was any difference in terms of intracellular RB51 survival inside infected WT or caspase-2 deficient macrophages. Our study showed that the *casp2*^−/−^ BMDMs were able to kill RB51 just as efficiently as WT BMDM (without statistically significant difference) (Figure S1C). Therefore, any differences in initiating cell death were not likely due to a lack of infectivity or bacterial killing in *casp2*^−/−^ BMDMs.

Since caspase-2 is required for RB51 induced cell death, we assessed which PCD pathway caspase-2 was mediating RB51-induced cell death. Inhibition of caspase-3 and/or caspase-8 activity led to a decrease in cell death, however, it was not as significant as caspase-2 deficiency (Table 1). Inhibition of caspase-3 or -8 in RB51-infected macrophages resulted in 57.3 ± 2.1% and 65.7 ± 3.7% cell death, respectively (*P* <0.001) (Table 1). Inhibition of both caspase-3 and -8 led to only 36.3 ± 2.5% cell death, suggesting the two types of inhibitions are likely additive but may also be synergistic. These data suggest that RB51-induced cell death may involve the apoptotic pathways associated with caspase-3 and caspase-8.

**Table 1:** **Cell death measurements in caspase-2 deficient and caspase-3 and -8 inhibited macrophages**.

**Condition**	**% Cell death (±*SD*)**
Untreated	0.7 ± 0.9
WT BMDMs	84.7 ± 1.7
Casp2KO BMDMs	12.7 ± 2.5
Z-DEVD-FMK (Casp3 inhibitor)	57.3 ± 2.1
Z-IETD-FMK (Casp8 inhibitor)	65.7 ± 3.7
Z-DEVD-FMK + Z-IETD-FMK	36.3 ± 2.5

Percent ± SD of n ≥ 3 independent experiments. Cells were counted in randomly selected fields of 100 cells. All conditions except untreated where in the presence of RB51 (MOI 200).

**Figure 1:**
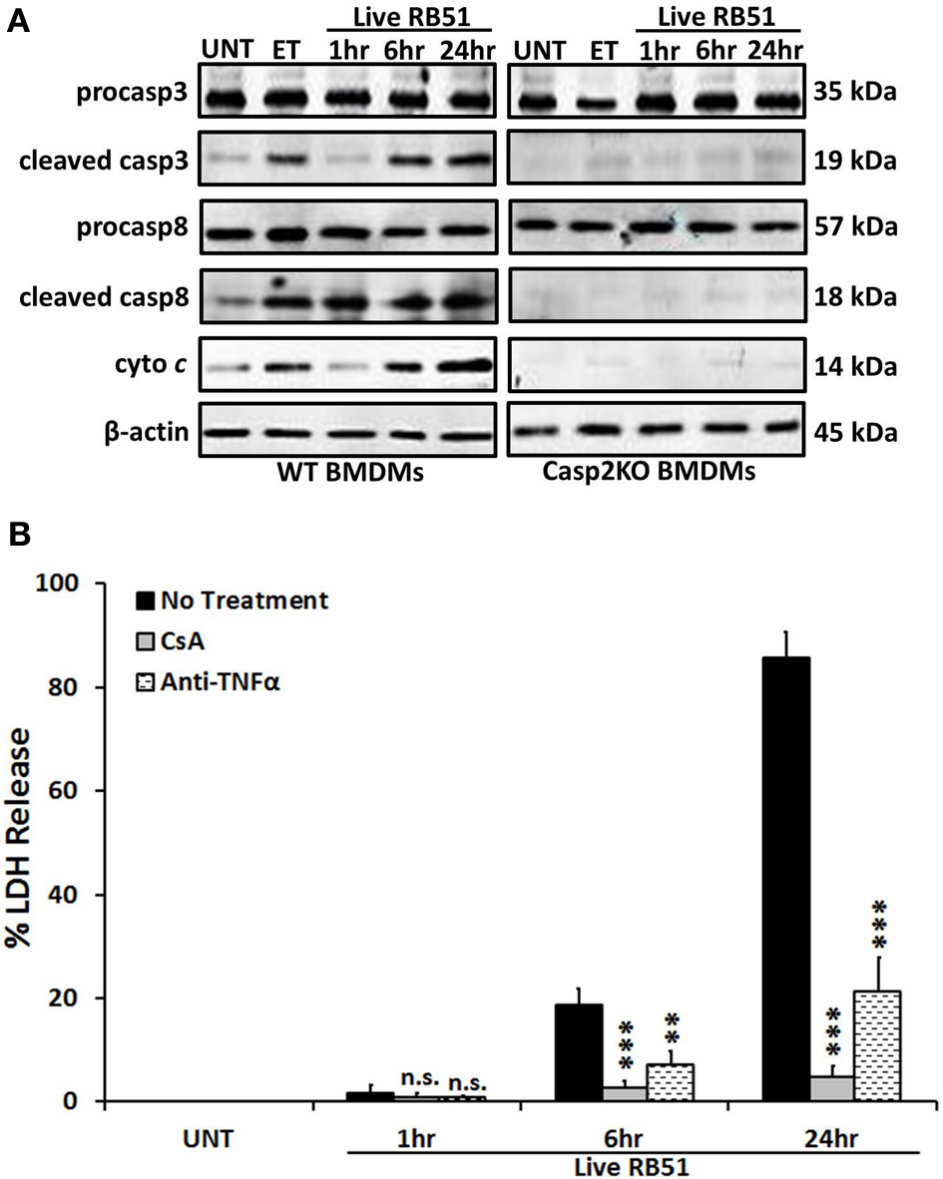
**Caspase-2 drives both the intrinsic and extrinsic cell death pathways. (A)** caspase-3 and -8 cleavage (activation) as well as cytochrome *c* (cyto *c*) release in Live RB51-infected WT and *casp2*^−/−^ BMDMs. β-actin served as a loading control. UT and ET represent untreated and etoposide (25 μM, 6 hr treatment), respectively. Immunoblots are representatives of *n* ≥ 3 independent experiments. **(B)** LDH release in Live RB51-infected RAW264.7 macrophages in the presence of cyclosporin A (CsA; inhibitor of mitochondrial permeability transition pore, 10 μM) and Anti-TNFα (10 μ g/mL). Error bars represent mean ± *SD* of *n* ≥ 3 independent experiments. ^**^p < 0.001 and ^***^p < 0.0001, Student's *t*-test. n.s. = not significant. Immunoblots are representatives of *n* ≥ 3 independent experiments.

Classically, caspase-3 and caspase-8 are linked to intrinsic (intracellular signal driven) and extrinsic (death receptor driven) apoptotic pathways, respectively (Elmore, 2007). Both pathways are linked to caspase-2 by mediating mitochondrial cytochrome *c* release and caspase-8 activation (Lin et al., 2004; Upton et al., 2008). To assess if caspase-2 regulated these pathways during RB51 infection, we investigated caspase-3 and caspase-8 activation in WT and *casp2*^−/−^ BMDMs by measuring cleavage. The cleavage of both caspase-3 and caspase-8 was abolished in RB51-infected *casp2*^−/−^ BMDMs (Figure 1A). Inhibition of caspase-3 and -8 did not affect caspase-2 activation (Figure S2). Previous studies illustrated that both the intrinsic and extrinsic cell death pathways can propagate signaling by inducing mitochondrial dysfunction, which eventually leads to cell death. Therefore, we investigated if caspase-2 mediated mitochondrial dysfunction in RB51-infected macrophages. Previously we observed that in RB51-infected macrophages, mitochondrial membrane potential decreased over time, suggesting that mitochondrial cytochrome *c* release (a marker of mitochondrial dysfunction) occurs. We found that in WT BMDMs, cytochrome *c* release increased throughout infection, however, in *casp2*^−/−^ BMDMs cytochrome *c* release was abolished (Figure 1A).

To assess if mitochondrial dysfunction contributed to RB51-induced cell death, the cytochrome *c* release was blocked with cyclosporin A (CsA) in RB51-infected RAW264.7 (murine) macrophages. CsA prevents opening of the mitochondrial permeability transition pore (MPTP), a pore responsible for the release of mitochondrial contents such as cytochrome *c* (Handschumacher et al., 1984). In the presence of CsA, cell death was significantly reduced (*p* < 0.0001) in RB51-infected macrophages (Figure 1B). Seeing that mitochondrial dysfunction occurs and both caspase-3 and caspase-8 are activated, we explored if RB51-induced cell death was acting through the extrinsic pathway. Extrinsic or death receptor mediated cell death can be activated by Fas ligand (FasL) and TNFα. Since live attenuated *B. suis* strain VTRS1 induces a proinflammatory response (Chen et al., 2011), we investigated if TNFα played a role in mediating RB51-induced cell death. We treated RAW264.7 macrophages with anti-TNFα and assessed cell death via LDH release. Anti-TNFα treatment led to a decrease in LDH release when compared to untreated RB51-infected RAW264.7 macrophages (Figure 1B, 85.5 ± 5.1% vs. 21.4 ± 6.5%, respectively). These observations suggest that caspase-2 drives the extrinsic cell death pathway in RB51-infected macrophages, and the TNFα cytokine that triggers the extrinsic cell death pathway is likely secreted from RB51-infected macrophages. It is also possible that the production of internal TNFα from a macrophage cell plays an important role of the PCD of the same macrophage cell.

### Caspase-2 regulates caspase-1 activation and IL-1β production in RB51-infected macrophages

**Figure 2:**
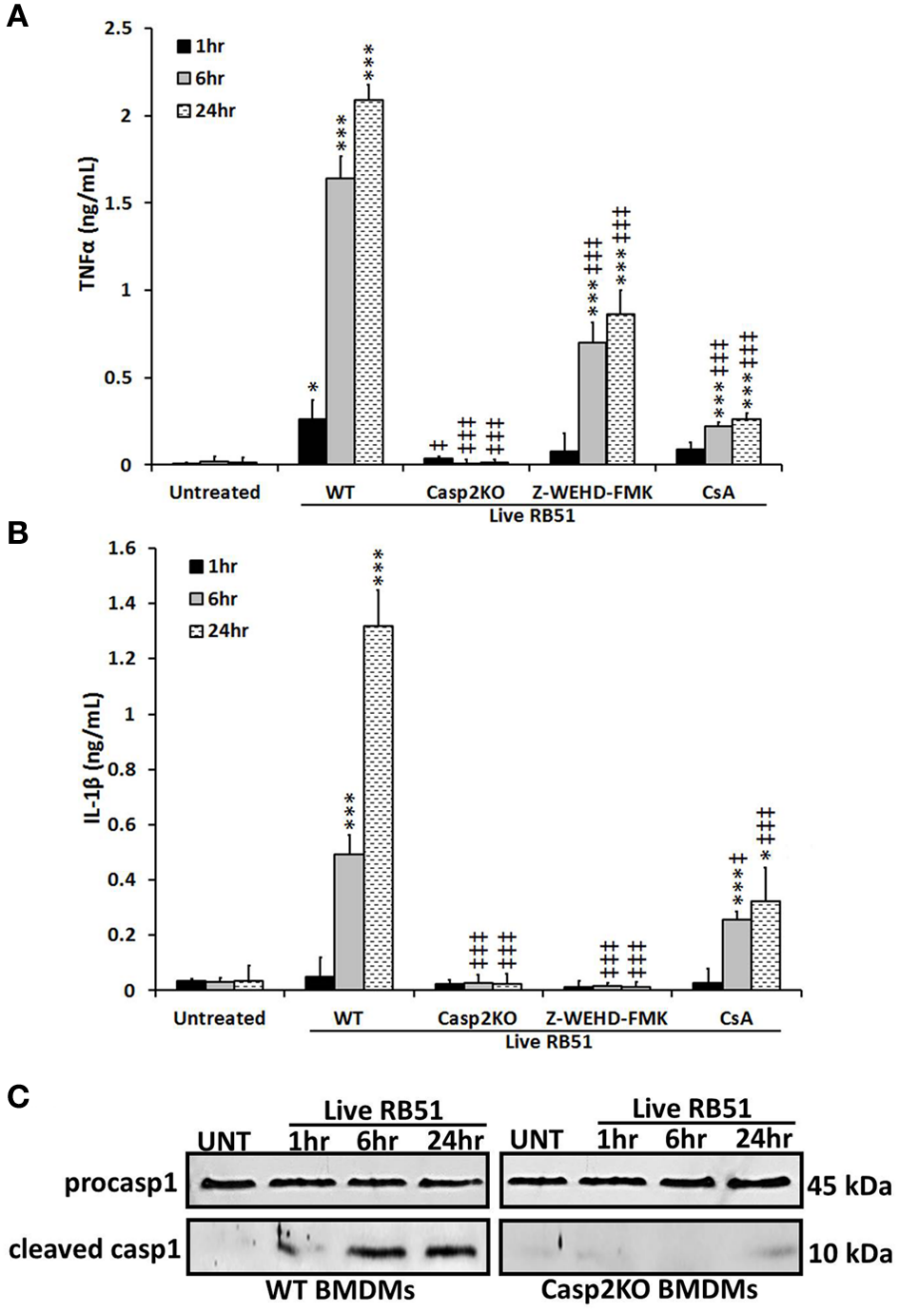
**Caspase-2 drives proinflammatory responses in RB51-infected macrophages. (A)** TNFα and **(B)** IL-1β levels in WT, casp2^−^/^−^, CsA (cyclosporin A, inhibitor of mitochondrial permeability transition pore, 10 μM) and Z-WEHD-FMK (caspase-1 inhibitor, 20 μM) treated BMDMs. Error bars represent mean ± *SD* of *n* ≥ 3 independent experiments. ‡, ^*^p < 0.01 and ‡‡‡, ^***^p < 0.0001, Student's *t*-test. (^*^, ^***^) and (‡, ‡‡‡) represent comparison to untreated and WT + Live RB51, respectively. **(C)** caspase-1 cleavage (activation) in Live RB51-infected WT and casp2^−^/^−^ BMDMs. UT represents untreated (negative control). Immunoblots are representatives of *n* ≥ 3 independent experiments.

Studies have illustrated that a proinflammatory response can be the trigger or product of cell death (Elmore, 2007; Bergsbaken et al., 2009). In addition to trigger extrinsic apoptotic cell death pathway, TNFα is also an important proinflammatory cytokine. Since TNFα played a role in RB51-induced cell death, we assessed if caspase-2 mediated TNFα production. Over time, TNFα levels increased in RB51-infected WT BMDMs. However, in *casp2*^−/−^ BMDMs, TNFα was reduced to untreated levels (Figure 2A). In addition, CsA treatment led to a decrease in TNFα production.

Another cytokine associated with proinflammatory cell death is IL-1β. During pyroptosis, caspase-1 processes IL-1β and aids in its secretion. Interestingly, caspase-2 contains a CARD domain and has been shown to mediate caspase-1 activation during *Salmonella* infections (Jesenberger et al., 2000). Similar to TNFα, IL-1β levels increased above untreated levels starting at 6 h.p.i., however, in *casp2*^−/−^ and caspase-1 inhibited BMDMs, IL-1β production was abolished (Figure 2B). In the presence of CsA, IL-1β levels were significantly reduced in RB51-infected macrophages. A decrease in IL-1β levels in *casp2*^−/−^ BMDMs suggested that either caspase-2 regulates caspase-1 activation or caspase-2 is directly responsible for the processing of IL-1β. To evaluate these two possibilities, caspase-1 cleavage in RB51-infected WT and *casp2*^−/−^ BMDMs was measured. RB51 induced caspase-1 cleavage in WT BMDMs starting at 6 h.p.i., however, cleaved caspase-1 was absent in *casp2*^−/−^ BMDMs (Figure 2C). These data suggest that caspase-2, via mitochondrial dysfunction, mediates caspase-1 activation and IL-1β production in RB51-infected macrophages.

### Pore formation is not required for RB51-induced cell death

**Figure 3:**
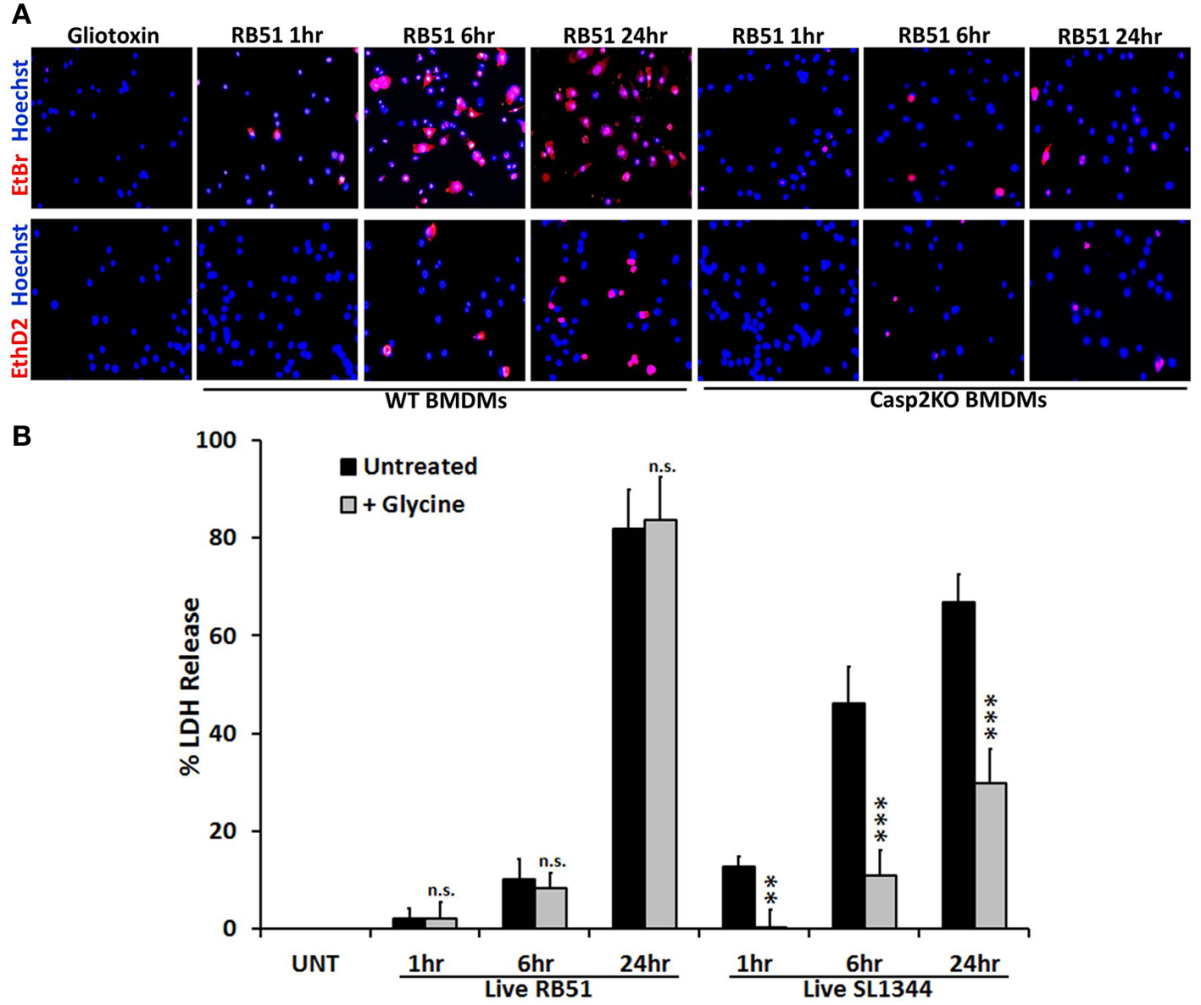
**Pore formation does not contribute to RB51-induced cell death. (A)** WT and casp2^−/−^ BMDMs treated with Gliotoxin (10 μM, 6 h treatment) or infected with RB51 were stained with the membrane permeable dye Hoechst 33342 (blue) and the membrane impermeant dyes (red), EtBr (MW 394) or EthD2 (MW 1293). Adherent cells were visualized by fluorescence microscopy (100x). Images are representatives of n ≥ 3 independent experiments. **(B)** LDH release in Live RB51 and SL1344-infected RAW264.7 macrophages in the absence or presence of glycine (5 mM). Error bars represent mean ± *SD* of *n* ≥ 3 independent experiments. ^**^p < 0.001 and ^***^p < 0.0001, Student's *t*-test. n.s. = not significant.

Caspase-1 activation and IL-1β production are key indicators of pyroptosis. During pyroptosis, pores form in the plasma membrane, leading to a change in ionic gradient and water influx. The consequence of the pores is cell swelling, due to water influx, and eventually cell lysis. *Salmonella enterica* serovar Typhimurium SL1344 is a pathogen known for inducing pyroptosis in macrophages (Fink and Cookson, 2006). We hypothesized that RB51 induced pore formation in macrophages. To test this, SL1344- and RB51-infected RAW264.7 macrophages were separately stained with two membrane impermeant dyes, ethidium bromide (EtBr, 394 Da) and the larger sized ethidium homodimer-2 (EthD2, 1293 Da). Uptake of EtBr and EthD2 would represent discrete pore formation and loss of plasma membrane integrity, respectively. Gliotoxin (apoptosis inducer) treated RAW264.7 macrophages excluded both impermeant dyes (Figure 3A), indicative of an intact plasma membrane. In SL1344- and RB51-infected WT BMDMs, uptake of EtBr began at 1 h.p.i. and increased throughout infection (Figure 3A). Uptake of EthD2 was significantly less than EtBr and did not occur until 6 h.p.i. and 24 h.p.i. in RB51- and SL1344-infected macrophages, respectively (Figure 3A and Figure S3A). Since pore formation has been shown to be dependent upon caspase-1, it was not surprising to see that uptake of both EtBr and EthD2 were abolished in RB51-infected *casp2*^−/−^ and caspase-1 inhibited WT BMDMs (Figure 3A and Figure S3B).

We further assessed if pore formation in RB51-infected macrophages contributed to RB51-induced cell death. RB51-infected macrophages were treated with glycine, a cytoprotective agent. Glycine prevents cell death by stabilizing the ion gradient (Frank et al., 2000). Glycine treatment decreased SL1344-induced macrophage cell death, however, no effect was seen with RB51 (Figure 3B). These findings demonstrate that although pore formation occurs in RB51-infected macrophages, it is not the cause of the RB51-induced macrophage cell death.

### Caspase-2 mediates cell death in other contexts

**Figure 4:**
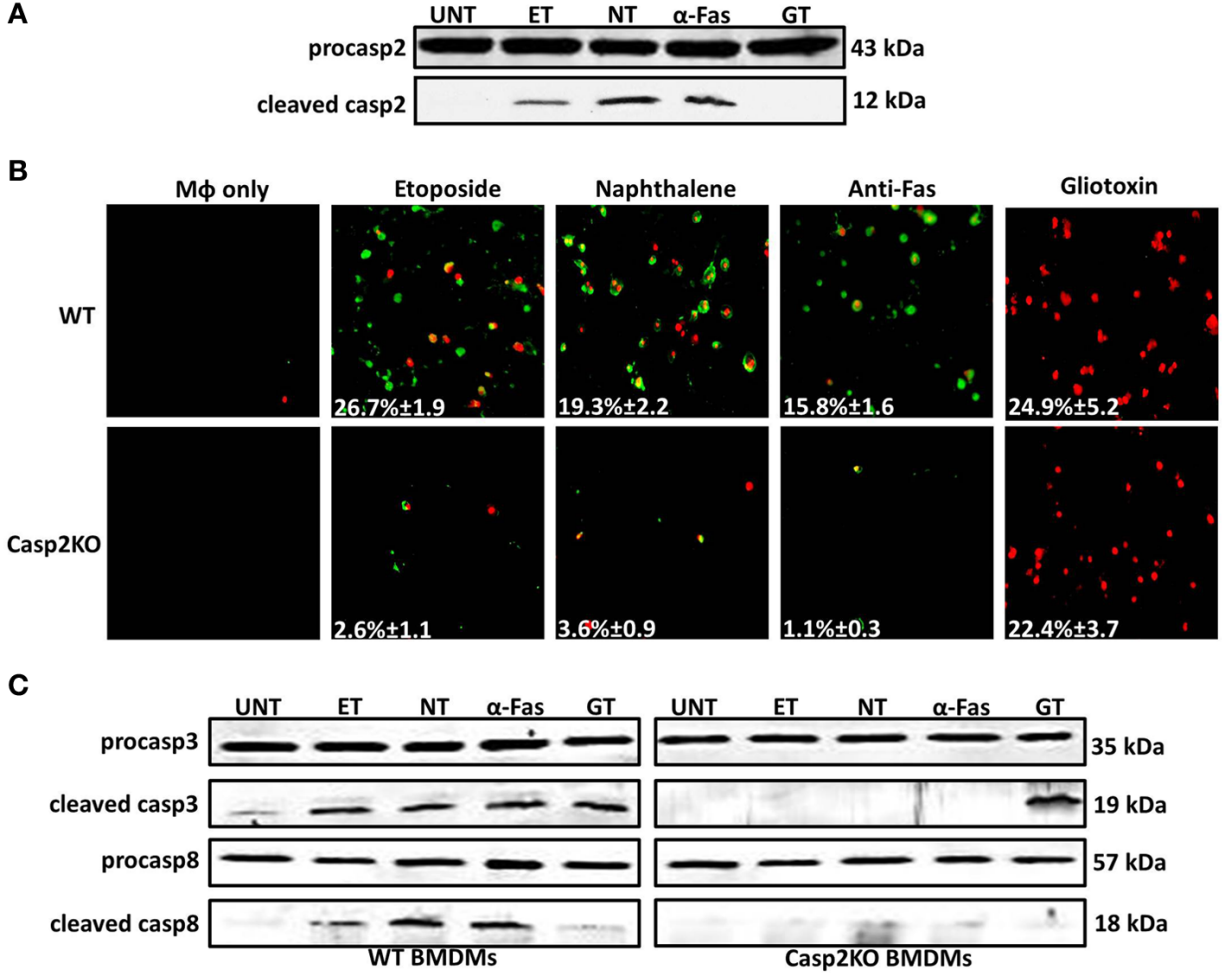
**Caspase-2 mediates caspase-3 and -8 activation in other contexts. (A)** Caspase-2 cleavage (activation) in Live RB51-infected RAW264.7 macrophages. UT, ET, NT, α-Fas, and GT represent untreated, etoposide (25 μM, 6 h treatment), Naphthalene (100 μM, 6 h treatment), Anti-Fas (1 μg/mL, 6 h 4 treatment), and Gliotoxin (10 μM, 6 h treatment), respectively. **(B)** ET, NT, α-Fas, GT treated WT and casp2^−^/^−^ BMDMs were stained with Annexin V/propidium iodide (PI). Adherent cells were visualized by fluorescence microscopy (100x). Cells were counted in randomly selected fields of 100 cells. Images are representatives of *n* ≥ 3 independent experiments. **(C)** Caspase-3 and −8 cleavage (activation) in ET, NT, α-Fas, GT treated WT and casp2−/− BMDMs. Immunoblots are representatives of *n* ≥ 3 independent experiments.

Since caspase-2 can regulate cell death in the context of a *Brucella* infection, we investigated if caspase-2 also plays a critical role in the presence of different cell death chemical inducers—etoposide, naphthalene, anti-Fas, and gliotoxin. Etoposide and naphthalene are DNA damaging agents that trigger mitochondrial ROS production, inducing cell death via the intrinsic pathway (Gorman et al., 1997; Bagchi et al., 1998, 2001; Pham and Hedley, 2001). Treatment with anti-Fas antibody mimics activation of death receptor signaling by FasL through the extrinsic pathway. (Aggarwal and Gupta, 1999). Gliotoxin mediates Bak activation leading to cytochrome *c* release from the mitochondria and cell death (Pardo et al., 2006). To assess the role of caspase-2 in the presence of these chemical inducers, we first assessed caspase-2 activation. In macrophages treated with etoposide, naphthalene, and anti-Fas, caspase-2 was activated. However, gliotoxin did not induce caspase-2 activation (Figure 4A). Next, we investigated if caspase-2 mediated cell death in the presence of these inducers via Annexin V/PI staining. All four chemicals induced cell death after 6 h of treatment. The caspase-2 deficiency led to a significant decrease in cell death in etoposide, naphthalene, and anti-Fas treated but not gliotoxin treated macrophages (Figure 4B).

Knowing the mechanism by which these chemical inducers mediate cell death, we assessed whether caspase-3 and -8 activation occurred and whether caspase-2 mediated their activation as seen in RB51-infected macrophages. Caspase-3 was activated in etoposide, naphthalene, anti-Fas, and gliotoxin treated WT BMDMs (Figure 4C). Caspase-3 activation was abolished in *casp2*^−/−^ BMDMs treated with etoposide, naphthalene, and anti-Fas but not gliotoxin. Caspase-8 activation was observed in etoposide, naphthalene, and anti-Fas treatment yet absent during gliotoxin treatment (Figure 4C). Similar to caspase-3, caspase-8 activation was abolished in etoposide, naphthalene, anti-Fas treated *casp2*^−/−^ BMDMs. These data indicate that caspase-2 can exhibit a crucial role in mediating cell death initiated by other stimuli besides microbial infection.

## Discussion

Here we report that RB51-induced cell death is driven by the caspase-2/mitochondrial axis. Our results demonstrated that RB51-infected macrophages induce the caspase-2 activation, and the activated caspase-2 mediates caspase-1, -3, and -8 activations, and TNFα and IL-1β secretions. Mitochondrial release of cytochrome *c* was associated with caspase-3 activation and contributed to RB51-induced cell death. Although pores formed in the plasma membrane upon infection, they did not contribute to cell death. In addition to cell death, we showed that the RB51-induced proinflammatory response was mediated by caspase-2 and mitochondrial dysfunction. In addition to microbial infections, caspase-2 also demonstrated a contributing role in mediating cell death with different cell death inducers.

Rough *Brucella* species are known to induce PCD (Freeman et al., 1961; Fernandez-Prada et al., 2003; Pei et al., 2006) as well as a robust immune response (Rittig et al., 2003). Here we show that proinflammatory cytokine TNFα plays a critical role in RB51-induced macrophage cell death, however, TNFα appears not to play an important role in macrophage cell death induced by the infection of strain CA180, another *B. abortus* rough strain (Pei et al., 2006). Although both RB51 and CA180 share a rough *Brucella* lipopolysaccharide (LPS) phenotype (i.e., containing deficient O antigen on their LPS), the LPS phenotype patterns in these two strains are likely different. CA180 has a truncated core polysaccharide due to a mutation in the phosphomannomutase gene (Allen et al., 1998). Phosphomannomutase is needed to the elongation of the polysaccharide core. The polysaccharide core has been shown to elicit cytokine production, any changes (e.g., shortening) can decrease cytokine production (Otterlei et al., 1993; Berntzen et al., 1998). The rough phenotype of RB51 is primarily due to the mutation of a gene *wboA* that encodes for a glycosyltransferase required for O-side chain sysnthesis (Vemulapalli et al., 1999). Additional mutation also exists in RB51 (Marianelli et al., 2004). Rough mutants with different gene mutations are known to possibly induce different phenotypes (Gonzalez et al., 2008). Therefore, the different gene mutations in RB51 and CA180 may explain the difference in the role of TNFα in the induced macrophage cell death.

Although caspase-2 has been linked to many different cellular processes, caspase-2 is still considered primarily a proapoptotic caspase. Here we show that caspase-2 can mediate IL-1β production, as well as caspase-1 activation. In addition to *Brucella* infection, caspase-2 has also been shown to regulate caspase-1 activation in *Salmonella* infected macrophages. We speculate that caspase-2 might regulate caspase-1 by interacting with the inflammasome complexes responsible for caspase-1 activation or by inducing an inflammasome trigger (e.g., ER stress, mitochondrial DNA release, or ROS production). Caspase-1 interacts with different inflammasome proteins and accessory proteins via its CARD domain. Inflammatory caspases (caspase-1, −4, −5, −11) interact with inflammasome and accessory proteins via their CARD domain. Caspase-2 also contains a CARD domain. Further investigation into whether caspase-2 can interact with different inflammasome components is needed. Another possible mechanism for caspase-2 regulation of caspase-1 activation is through mitochondrial dysfunction. As seen with CsA treatment, there was a sharp decrease in IL-1β production suggesting that the mitochondria can aid in inflammasome activation during RB51 infection. Recent studies have linked NLRP3 and AIM2 activation to mitochondrial dysfunction (Nakahira et al., 2011; Misawa et al., 2013; Subramanian et al., 2013). It is possible that caspase-2-induced mitochondrial dysfunction leads to release of mitochondrial danger associated molecular patterns (DAMPs). Mitochondrial DAMPs (e.g., mtDNA or cardiolipin exposure) can lead to NLRP3 and AIM2 inflammasome activation (Nakahira et al., 2011). Caspase-2 has been linked to mediating mitochondrial dysfunction—caspase-2 cleaves Bid leading to mitochondrial outer membrane pore formation and eventually release of mitochondrial content (e.g., cytochrome *c*). Whether caspase-2-mediated release of mitochondrial DAMPs is the mechanism by which the inflammasome and caspase-1 activation occurs remains to be elucidated.

The caspase-2-mediated cell death pathway seen in RB51-infected macrophages differs from classical apoptosis, caspase-1-mediated pyroptosis, and necroptosis (Table 2). RB51-induced cell death is not mediated by caspase-1. Our data suggest that RIP1 and RIP3 (mediators of necroptosis) are not active because both caspase-2 and -8 activated by RB51 infection can cleave RIP1 and RIP3—the cleavage of these two prevents necroptosis from occurring (Lin et al., 1999; Lamkanfi et al., 2005). In addition, RB51-infected macrophages secrete proinflammatory cytokines starting at 6 h.p.i., a characteristic absent in apoptosis. Caspase-1 is active and contributes to IL-1β production. However, unlike the phenomenon seen in caspase-1-mediated pyroptosis, neither caspase-1 nor the pore in the plasma membrane contributes to cell death. Our data suggest that RB51-induced caspase-2-mediated cell death does not fall into any of the three classical PCD pathways. The term “pyroptosis” was originally coined to indicate proinflammatory (=“pyro”) PCD (“=ptosis”) (Bergsbaken et al., 2009). Owing to the common feature of caspase-mediated proinflammatory cell death between caspase-1-mediated pyroptosis and caspase-2-mediated cell death, previously we tentatively labeled the caspase-2-mediated proinflammatory cell death as “caspase-2-mediated pyroptosis” (Chen et al., 2011). Considering that current study also clearly shows that caspase-2 also mediates apoptosis-like features (e.g., caspase-3 and -8 activation and loss of mitochondrial membrane potential) (Table 2), caspase-2 appears to mediate a hybrid cell death that includes partial features of both non-proinflammatory apoptosis and caspase-1-mediated proinflammatory pyroptosis. Although distinct, caspase-2-mediated cell death and necroptosis appear to also share some similarities as well (Table 2).

**Table 2:** **Comparison of caspase-2-mediated cell death to classical apoptosis and pyroptosis**.

	**Apoptosis**	**Casp-1 pyroptosis**	**Necroptosis**	***Casp2*-mediated cell death**
Plasma membrane pore formation	−	+	−	+
Loss in plasma membrane integrity	−	+ (swelling = lysis)	+	+ (6 and 24 h.p.i.)
Loss of mitochondrial membrane potential	+	−	+	+
Cytochrome *c* release	+	−	+	+
Proinflammatory	−	+	+	+
Caspase-1 mediated	−	+	−	−
Caspase-2 mediated	+/−	−	−	+
Caspase-3 activation	+	−	−	+
Caspase-8 activation	+/− (extrinsic)	−	−	+
ROS production	+	−	+	+
Release of DAMPs	−	+	+	+
RIP1/3-mediated	−	−	+	?

+, occurs; −, does not occur; +/−, may occur but not required; ?, unknown.

In addition to RB51, rough attenuated *B. abortus* strain RA1 (Chen and He, 2009) and *B. suis* strain VTRS1 also induces caspase-2-mediated macrophage cell death (Chen et al., 2011). However, their parent WT strains 2308 and 1330 do not induce macrophage cell death. Our recent study found that caspase-2 also mediates PCD of dendritic cells infected with RB51 and its WT strain 2308 (Li and He, 2012). The induction of caspase-2-mediated cell death in strain 2308-infected dendritic cells but not in strain 2308-infected macrophages facilitates the survival of the virulent strain inside macrophages and avoidance of *Brucella* antigen presentation inside dendritic cells (Li and He, 2012). It is likely that such caspase-2-mediated host cell death is also induced or inhibited by other infectious microbes, such as live attenuated vaccine strains or virulent pathogens of intracellular bacteria and viruses.

Our studies on the relationship between caspase-2 and cell death induced by different cell death inducers further illustrate the critical and conserved role and the mechanism of caspase-2 in triggering PCD. Both etoposide and naphthalene are DNA damage inducers, a stimulus known for activating caspase-2. Interestingly, both etoposide and naphthalene induced caspase-8 activation. Previous studies have illustrated a connection between DNA damage, caspase-8, and caspase-2. Both caspase-2 and caspase-8 can be activated by p53 (Seth et al., 2005; Liu et al., 2011); this suggests that upon etoposide and naphthalene treatment, p53 activates caspase-2 leading to caspase-8 activation. Anti-Fas works through the extrinsic pathway and leads to cell death via caspase-8 dependent activation of caspase-3. Seeing that caspase-2 aided in caspase-8 activation (outside of DNA damage) clearly suggests that caspase-2 can act as an initiator caspase to mediate activation of other caspases. Whether caspase-2 mediates caspase-8 activation directly or indirectly remains unclear. Gliotoxin induced cell death via the intrinsic pathway. Only caspase-3 was activated in gliotoxin-treated macrophages. Cytochrome *c* can aid in caspase-2 activation, however, (Slee et al., 1999), in the context of gliotoxin intoxication this was not the case because caspase-2 activation did not occur. Gliotoxin acts on Bak and its downstream target t-Bid [a known target cleaved by caspase-2, (Upton et al., 2008)]; therefore, it is possible that gliotoxin short-circuits the classical intrinsic pathway and does not require signaling components upstream of the mitochondria. In the case of etoposide and naphthalene, the data suggest that caspase-2 regulates caspase-3 activation via the mitochondria. Caspase-3 is activated via the apoptosome, a multiprotein complex dependent upon cytochrome *c* release. Caspase-2 was previously reported to act upstream of caspase-8 during ceramide-induced mitochondrial apoptosis in T cells (Lin et al., 2004). It appears that the caspase-2 regulation of caspase-3 and -8 can occur in different cell types with different treatments, so this type of regulation is neither cell specific nor context specific. These observations suggest that caspase-2 can play a critical role in initiating PCD.

The caspase-2-mediated cell death pathway is likely critical to microbial pathogenesis and host immunity. In the context of RB51, cell death and the proinflammatory response may have synergistic effects on host immune responses. Cell death may result in the exposure of RB51 to a more hostile extracellular environment (as seen in pyroptosis and necroptosis). In addition, neighboring macrophages and dendritic cells may recognize processed RB51 antigens leading to cross priming of CD8^+^ T cells (important for RB51-induced protective immunity). We recently showed that RB51 induced cell death in bone marrow derived dendritic cells and aided in maturation as well as priming of T cells—all of which were dependent upon caspase-2 (Li and He, 2012). These observations demonstrate the importance for caspase-2, as well as cell death, in initiating the immune response. Utilizing this PCD pathway may ensure that the host triggers a potent immune response. Prevention of this pathway may aid in enhancing survival of virulent *Brucella* in macrophages. Our previous work suggested prevention—virulent strain *B. abortus* S2308 did not induce caspase-2 activation nor cytochrome *c* release in infected macrophages (Chen and He, 2009).

Caspase-2 is also implicated in other processes (cancer regulation and metabolism) and may take on a regulatory role in these processes as well. We have made an original observation that caspase-2 plays a non-redundant role in triggering the proinflammatory cell death of RB51-infected macrophages and in macrophages treated with various drugs. After the evolution of complex caspase-cascade cell death signaling pathways in advanced animals, it is suggestive that the protein functions of the highly conserved caspase-2 have been preserved during evolution and serve as safeguards to regulate various cell death pathways. It is likely that intracellular pathogens with similar lifestyles to *Brucella* (e.g., *Salmonella*, *Mycobacterium*, *Listeria*, *Francisella*, and *Legionella*) may utilize caspase-2 during infection. Further understanding of caspase-2-mediated pyroptosis can aid in supplying a blueprint for effective brucellosis vaccines (both animals and humans) as well as effective therapeutics against cancers and other diseases.

## Materials and methods

### Mice

The caspase-2 knockout (Casp2KO) mice were originally generated by Junying Yuan and kindly provided by Dr. Brian Herman of the University of Texas Health Science Center at San Antonio with Dr. Yuan's consent (Bergeron et al., 1998). The deletion inactivates both the long and short form of caspase-2. The mice were backcrossed with C57BL/6 once in the Unit for Laboratory Animal Medicine at the University of Michigan Medical School, and then used as founders. Casp2KO and WT C57BL/6 (Jackson) mice with similar ages were applied in the experiments. The University Committee on Use and Care of Animals (UCUCA) at the University of Michigan approved the protocol (#09695) to use mice for studies described here.

### Bacterial strains and reagents

RAW264.7 macrophages and bone marrow derived macrophages (BMDMs) were infected with *Brucella abortus* strain RB51 (from Dr. G. Schurig, Virginia Polytechnic Institute and State University) and *Salmonella typhimurium* SL1344 (from Mary O'Riordan, University of Michigan). The following inhibitors and inducers were used: CsA (Sigma-Aldrich), etoposide (Sigma-Aldrich), naphthalene (Sigma-Aldrich), Anti-Fas (BioVision), gliotoxin (-Aldrich), glycine (Sigma-Aldrich), Z-WEHD-FMK (Caspase-1 inhibitor, R&D Systems), Z-DEVD-FMK (Caspase-3 inhibitor, R&D Systems), Z-IETD-FMK (Caspase-8 inhibitor, R&D Systems), and anti-TNFα (mouse specific, BioVision).

The following antibodies were used: anti-cytochrome *c* (cat#:4272S, Cell Signaling), anti-caspase-3 (cat#: 9662S, Cell Signaling), anti-caspase-8 (cat#: 4927S, Cell Signaling), anti-caspase-2 (cat#: 3027-100, BioVision), anti-caspase-1 (cat#: sc-514, Santa Cruz), and anti-actin (cat#: MS1295P1, Thermo Scientific).

### Cell culture and infection

BMDMs were isolated from WT and *casp2*^−/−^ mice on a C57BL/6 background. Isolated BMDMs were differentiated in DMEM (GIBCO) supplemented with 20% heat-inactivated FBS (GIBCO), 1% L-glutamine (200 mM), 1% sodium pyruvate (100 mM), 0.1% β-mercaptoethanol (55 mM), and 30% L-929 fibroblast conditioned medium. BMDMs were cultured in non-TC treated plates, fed fresh media on Day 3, and harvested on Day 6. BMDMs were maintained at 37°C under 5% CO_2_.

Four million RAW264.7 macrophages and BMDMs were seeded in 6 well plates 18 h prior to infection. The following day, where indicated cells were pretreated with CsA (10 μM), Z-WEHD-FMK (20 μM), Z-DEVD-FMK (20 μM), Z-IETD-FMK (20 μM) and Anti-TNFα (10 μ g/mL) for 1 h prior to infection. Untreated and pretreated cells were infected with RB51 (MOI 200) or SL1344 (MOI 25) for 30 min, after which the inoculum was removed and cells were washed with PBS. Medium containing 50 μ g/ml of gentamicin was added to kill extracellular bacteria. To synchronize infection, cells were spun at 1200 rpm for 3 min after adding inoculum. Cells were treated with etoposide (25 μM), naphthalene (100 μM), anti-Fas (1 μ g/mL), and gliotoxin (10 μM) for 6 hr. At the indicated times, cells were lysed in buffer containing 1% NP-40 on ice for 15 min and spun at 13,000 rpm for 15 min to pellet the insoluble fraction. Soluble fractions were used for immunoblot assays.

### Immunoblot assay

Cytosolic extracts collected at various time points (1, 6, and 24 h pi) were separated by SDS-PAGE, transferred to PVDF membranes (Millipore), blocked with 5% milk in TBS-Tween20 (TBS-T), and incubated overnight at 4°C with primary antibodies stated above. Membranes were washed with TBS-T and incubated with secondary HRP conjugated to either goat anti-rabbit IgG (cat#: 12–348, Millipore) or goat anti-mouse IgG (cat#: 1034-05, Southern Biotech) at room temperature for 1 h. Bands were visualized using the ECL Western Blotting Substrate Kit (Pierce). Immunoblots in the figures are representative of *n* ≥ 3 independent experiments.

### Cytokine detection

Culture supernatants were collected at different time points (1, 6, and 24 h pi) from macrophages infected as described above. IL-1β and TNFα levels were determined by sandwich enzyme-linked immunosorbent assay (ELISA) according to manufacturer's instructions (BioLegend). A minimum of three technical replicates per experiment and three experimental replicates were analyzed for each condition.

### Cell death assay

RAW264.7 macrophages were seeded in 6 well plates at a concentration 9.6 × 10^4^ per well and infected with RB51 as stated above. Cells were stained with Annexin V and PI using the Annexin V-FLUOS staining kit (Roche Diagnostics Corporation). Cells were washed with PBS and incubated with the fluorescent dyes for 15 min in the dark at room temperature. Fluorescence was observed with a Nikon TK-2000-S microscope and photographed with a RT Slide Spot digital camera and QCapture Pro software. Uninfected macrophages served as negative controls.

### Ethidium bromide (EtBr) and ethidium homodimer-2 (EthD2) staining

Macrophages were grown in 6-well plates and infected as described above. At different time points post-infection, cells were washed with PBS (GIBCO) and stained with Hoechst 33342 (5 μ g/mL) and either EtBr or EthD2 (25 μ g/mL) according to the manufacturer's instructions. Cell were analyzed with a Nikon TK-2000-s microscope and photographed with a RT slide spot digital camera and QCapture Pro Software.

### Lactate dehydrogenase (LDH) release assay

Macrophages were seeded in 96-well plates and infected with RB51 or SL1344 as stated above. Supernatants were analyzed for the presence of LDH enzyme using the CytoTox-ONE™ Homogeneous Membrane Integrity Assay (Promega) as directed by the manufacturer's instructions. Percentage of LDH release was calculated as 100 × [(Experimental LDH Release—Culture Medium Background)/(Maximum LDH Release—Culture Medium Background)].

### Statistical analysis

All *p*-values were generated between identified samples using unpaired two-tailed Student's *t*-tests and represent analysis of ≥3 replicates per condition. ‡, ^*^p < 0.01 ‡‡, ^*^p < 0.001 and ‡‡‡, ^***^p < 0.0001.

## Author contributions

Denise N. Bronner and Yongqun He designed the experiments. Denise N. Bronner performed the experiments. Denise N. Bronner, Mary X. D. O'Riordan and Yongqun He analyzed the data. Yongqun He and Mary X. D. O'Riordan contributed reagents and materials. Denise N. Bronner and Yongqun He wrote the paper. All authors edited and approved the manuscript.

### Conflict of interest statement

The authors declare that the research was conducted in the absence of any commercial or financial relationships that could be construed as a potential conflict of interest.

## Supplementary meterial

Figure S1 **Caspase-2 mediates RB51-induced macrophage cell death. (A)** Annexin V/propidium iodide (PI) staining of RB51-infected WT and *casp2*^−/−^ BMDMs at 100X magnification. Images are representatives of *n* ≥ 3 independent experiments. **(B)** LDH release in Live RB51-infected WT and *casp2*^−/−^ BMDMs. Cells were counted in randomly selected fields of 100 cells. **(C)** CFU analysis of RB51 in WT and *casp2*^−/−^BMDMs, Error bars represent mean ± *SD* of *n* ≥ 3 independent experiments. ^**^*p* < 0.001 and ^***^*p* < 0.0001, Student's *t*-test. n.s. = not significant.

Figure S2 **Caspase-3 and -8 are involved in RB51-induced cell death. (A)** Caspase-2 cleavage (activation) in Live RB51-infected RAW264.7 macrophages with or without Z-DEVD-FMK (20 μM, Caspase-3 inhibitor). **(B)** Caspase-2 cleavage (activation) in Live RB51-infected RAW264.7 macrophages with or without Z-IETD-FMK (20 μM, Caspase-8 inhibitor). UNT and ET represent untreated and etoposide (25 μM, 6 h treatment), respectively. Immunoblots are representatives of *n* ≥ 3 independent experiments.

Figure S3 **Caspase-1 aids in RB51-induced pore formation**. RB51-infected RAW264.7 macrophages treated with Z-YVAD-FMK (20 μM, caspase-1 inhibitor) were stained with the membrane permeable dye Hoechst 33342 (blue) and the membrane impermeant dyes (red), EtBr (MW 394) or EthD2 (MW 1293). Adherent cells were visualized by fluorescence microscopy (100x). Images are representatives of *n* ≥ 3 independent experiments.
