# Supplementary material for: Caspase-2 mediates a Brucella abortus RB51-induced hybrid cell death having features of apoptosis and pyroptosis
Source: Front Cell Infect Microbiol. 2013 Nov 27;3:83. doi: 10.3389/fcimb.2013.00083 (PMC3842122; doi:10.3389/fcimb.2013.00083)
Supplement: Figure S1 — Caspase-2 mediates RB51-induced macrophage cell death. (A) Annexin V/propidium iodide (PI) staining of RB51-infected WT and casp2−/− BMDMs at 100X magnification. Images are representatives of n ≥ 3 independent experiments. (B) LDH release in Live RB51-infected WT and casp2−/− BMDMs. Cells were counted in randomly selected fields of 100 cells. (C) CFU analysis of RB51 in WT and casp2−/−BMDMs, Error bars represent mean ± SD of n ≥ 3 independent experiments. **p < 0.001 and ***p < 0.0001, Student's t-test. n.s. = not significant. [file DataSheet1.PDF]

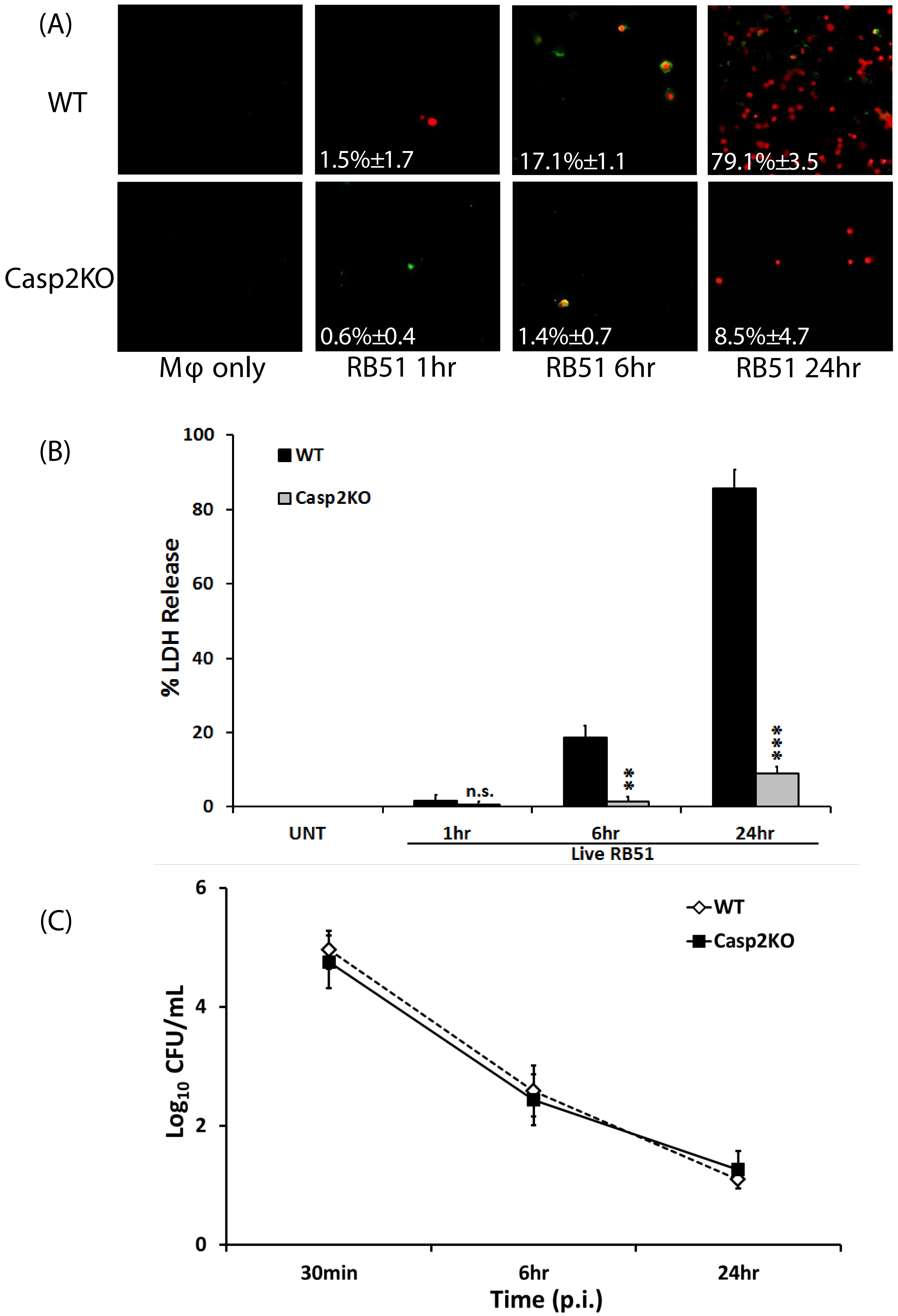

Supplemental Figure 1: Caspase-2 mediates RB51-induced macrophage cell death. (A) Annexin V/propidium iodide (PI) staining of RB51-infected WT and casp2$^{-/-}$ BMDMs at 100X magnification. Images are representatives of n≥3 independent experiments. (B) LDH release in Live RB51-infected WT and casp2$^{-/-}$ BMDMs. Cells were counted in randomly selected fields of 100 cells. (C) CFU analysis of RB51 in WT and casp2$^{-/-}$ BMDMs, Error bars represent mean ± SD of n≥3 independent experiments. **$p < 0.001$ and ***$p < 0.0001$, Student's t-test. n.s. = not significant.

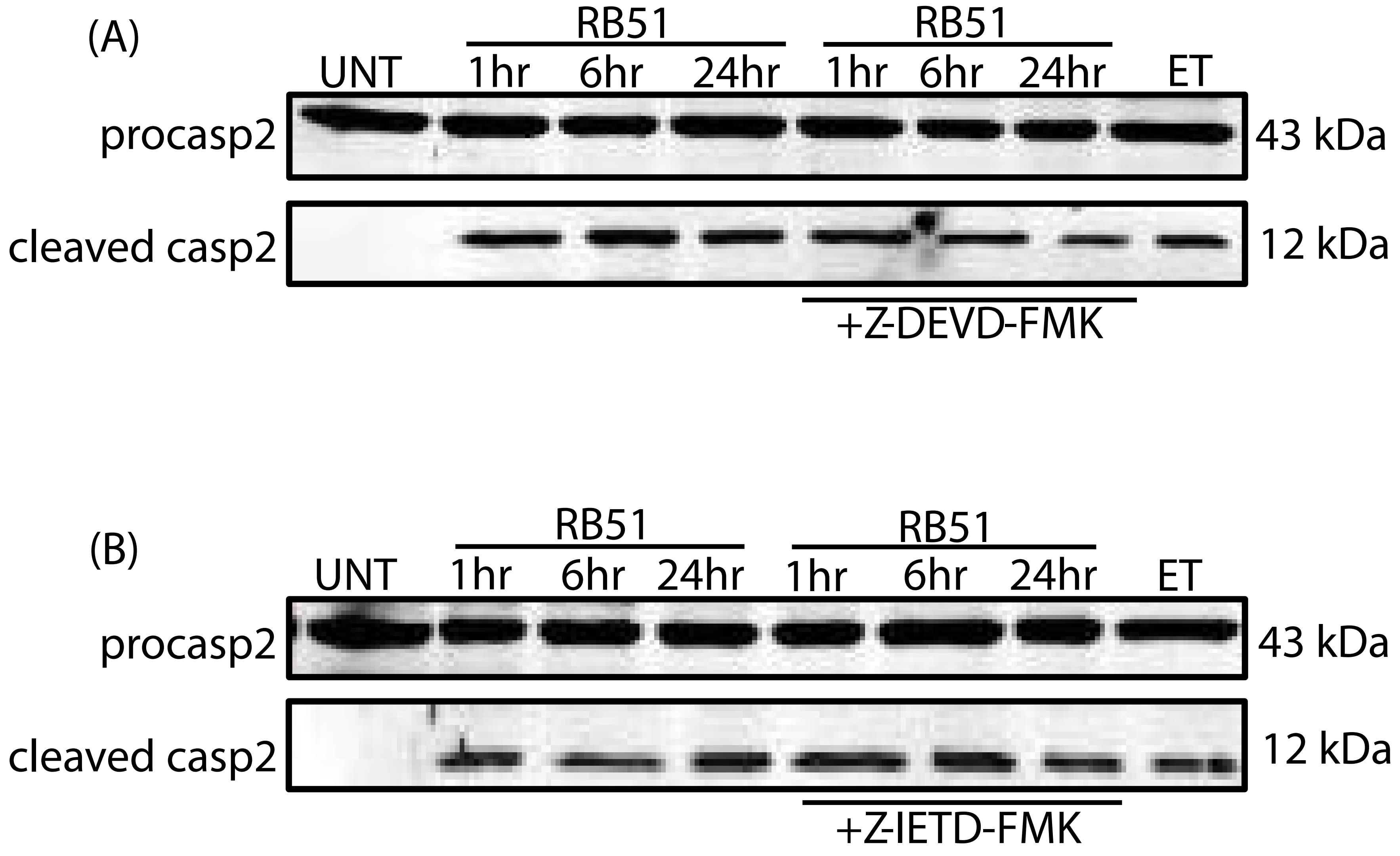

Supplemental Figure 2: Caspase-3 and -8 are involved in RB51-induced cell death. (A) Caspase-2 cleavage (activation) in Live RB51-infected RAW264.7 macrophages with or without Z-DEVD-FMK (20 µM, Caspase-3 inhibitor). (B) Caspase-2 cleavage (activation) in Live RB51-infected RAW264.7 macrophages with or without Z-IETD-FMK (20 µM, Caspase-8 inhibitor). UNT and ET represent untreated and etoposide (25 µM, 6hr treatment) respectively. Immunoblots are representatives of n≥3 independent experiments.

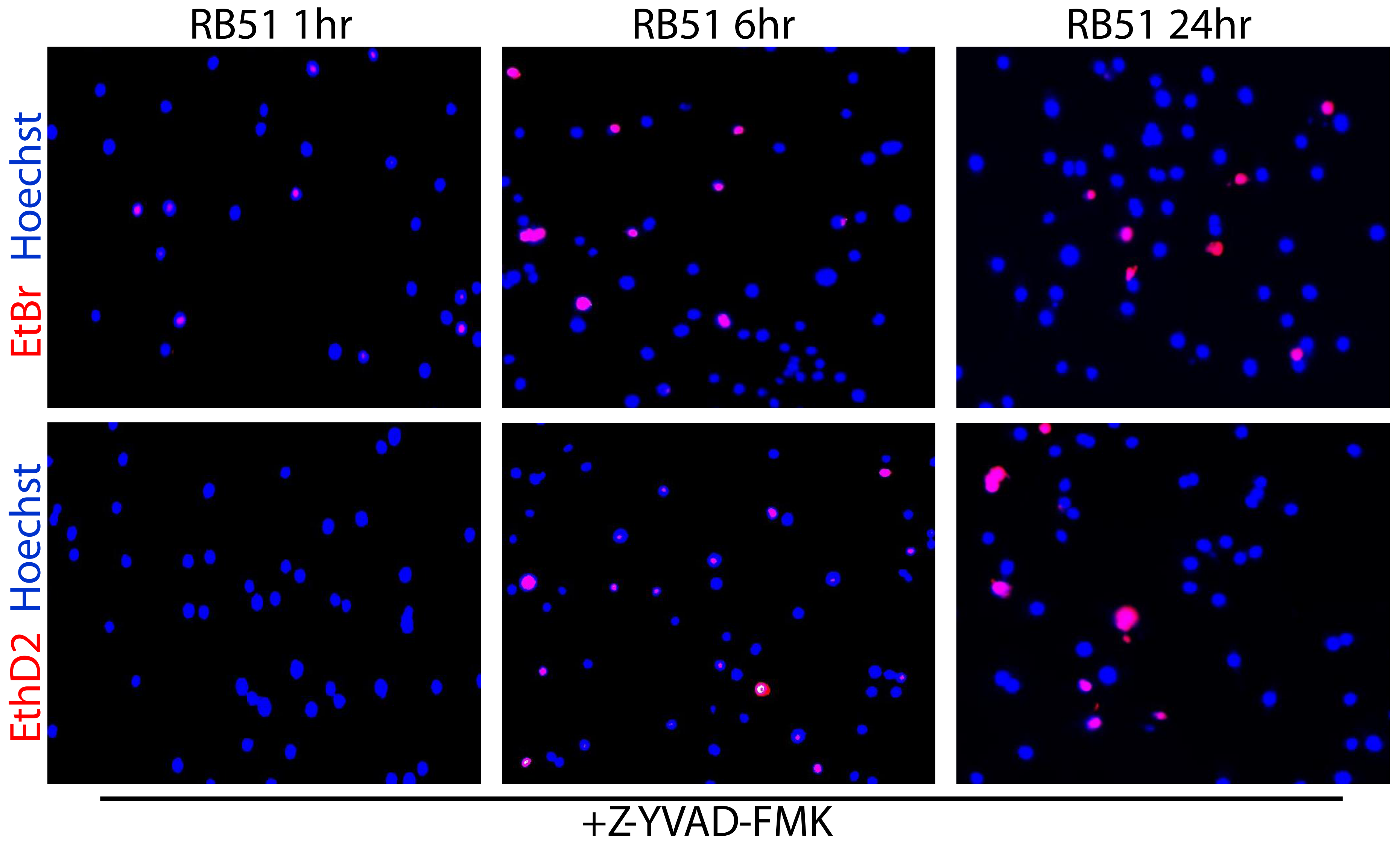

Supplemental Figure 3: Caspase-1 aids in RB51-induced pore formation. RB51-infected RAW264.7 macrophages treated with Z-YVAD-FMK (20 µM, caspase-1 inhibitor) were stained with the membrane permeable dye Hoechst 33342 (blue) and the membrane impermeant dyes (red), EtBr (MW 394) or EthD2 (MW 1293). Adherent cells were visualized by fluorescence microscopy (100x). Images are representatives of n≥3 independent experiments.
